# The impact of TikTok short video factors on tourists’ behavioral intention among Generation Z and Millennials: The role of flow experience

**DOI:** 10.1371/journal.pone.0315140

**Published:** 2024-12-05

**Authors:** Congying Liu, Mingdi Jiang, Zulqarnain Arshad Muhammad

**Affiliations:** 1 School of Tourism, Hospitality and Event Management, Universiti Utara Malaysia, Sintok, Malaysia; 2 Forge Business School, College of Mobile Communication, Chongqing, China; 3 School of Business Management, College of Business, Universiti Utara Malaysia, Sintok, Malaysia; Sri Sivasubramaniya Nadar College of Engineering, INDIA

## Abstract

Tourism advertising and tourism promotion have over the years been the core functions of tourism departments and major tourist sites. In relation to the progressing development of new media, the mobile short-form videos, which are short, focused, and have an engaging content, appear to be a useful means of advertising tourist destinations. In the digital era, short videos have become a new communication tool between destinations and consumers. This current study, based on the S-O-R model and flow experience, investigated the psychological processes through which TikTok attributes and technology evoke flow and lead to tourists’ behavioral intention. Moreover, the TAM, i.e., PU and PEOU, as two technology factors, as well as three content attributes (entertainment, informativeness, and interactivity) were examined. The study utilized a quantitative approach and collected data from 412 respondents in China. The authors adopted the PLS-SEM method to confirm the directions hypothesized in this model. There are significant effects of PU, PEOU, and entertainment on flow experience (telepresence, time distortion, and focused attention). Interactivity impacts telepresence and time distortion, while informativeness impacts focused attention. Moreover, time distortion and focused attention impact tourists’ behavioral intention. The results highlight several limitations and offer implications for future research as well.

## Introduction

Currently, the usage of smart mobile devices and the emergence of 4G networks have contributed to the advancement of information technologies and helped people to express themselves with the help of social networks. This has led to the emergence of short tourism videos produced by users themselves. Due to advancement in internet technology, many people have subscribed to many social media sites, making it an effective channel of communication between the consumers and the marketers [1]. Being one of the most popular tools for internet communication, social media plays a crucial role in most of the social and economic practices in the real world. Short videos are another new phenomenon, which have gained significant ground in this digital environment. Specifically, short-form video platforms have become new trends for social media, and many age groups have started to adopt this tool. Understanding video content in destination-specific contexts and strategies from the perspectives of tourism destinations and content creators on these platforms, is relevant and essential. This, in turn, involves determining the attributes that can make such media more effective in reaching and setting the desired behaviors among the target groups.

Unlike other social media platforms, short-form videos are usually 15-seconds to one minute in length with different background music and special effects [2]. TikTok, also known as Douyin in China, is a representative short-form video platform with over 750 million daily active users [3]. Additionally, new types of applications (apps), like TikTok, Wechat or Kuaishou, have brought new kinds of mobile Internet as the primary means of a source and brand communication for users. At the same time, the search for information about a trip via these channels has also grown in popularity, and has quickly turned into a standard marketing method in the tourism sector. It is due to the versatile potential of the platforms with massive audience capability, opportunity for active interaction, huge number of emotional appeals, and practically, no expenses on advertising. These platforms do not only introduce tourist attractions to a large number of Internet-connected users, but also allow users share experiences, tastes and preferences of traveling, more so as these users continue to be enamored by short videos or films on traveling. The TikTok platform, first launched in 2016, has quickly emerged as the leading short video platform worldwide. In the context of tourism, short tourism TikTok videos play a significant role in various arenas, such as enhancing a destination’s image and improving tourism behavioral intention [4, 5].

**Fig 1:**
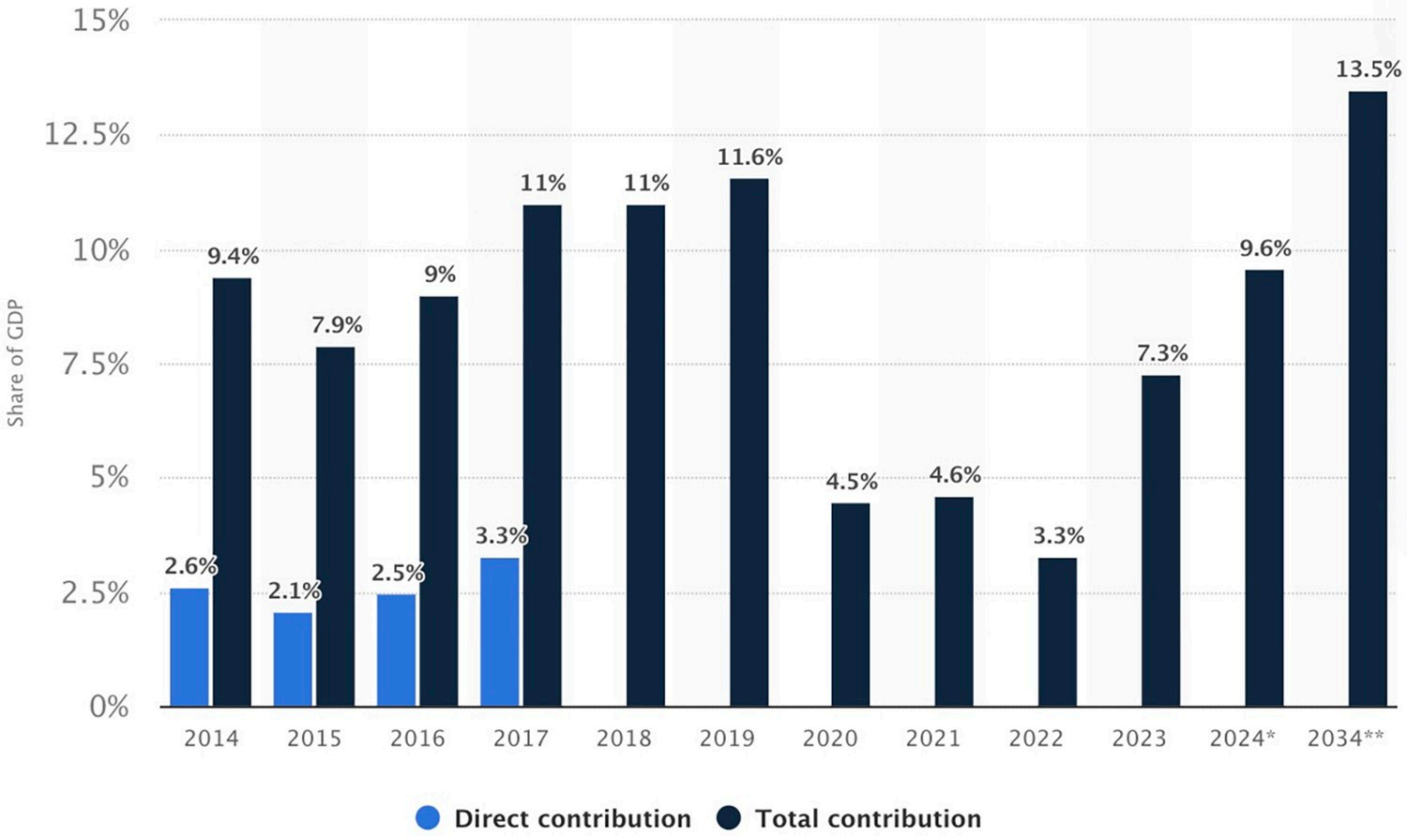
Travel and tourism industry’s share of GDP in China (2014 to 2022) with a 2024 forecast for 2034 (Source: Statista, 2024). https://www.statista.com/statistics/249794/contribution-of-chinas-travel-and-tourism-industry-to-gdp/.

The COVID-19 pandemic had a profound impact on the global economy, particularly on the tourism sector [6]. Based on public statistics data (Fig 1), the contribution of the tourism and travel industry to Gross Domestic Product (GDP) was 7.3% in 2023, a notable increase from 3.3% in 2022 [7]. However, it still lags behind the pre-COVID-19 percentages. Many local tourism government agencies are starting to use social media platforms to attract and encourage Chinese residents to travel. For instance, Xi’an has become an overnight famous destination due to the short TikTok video, known as “bowl-smashing wine” [8]. This successful example of Xi’an has inspired the local tourist destinations to start using short tourism videos as a marketing tool to catch people’s attention [9]. Unlike other social media platforms, short videos are typically more flexible and accessible to a broader range of users. TikTok boasts a unique algorithm that tailors content recommendations to each user, enhancing the platform’s user experience [10].

Young demographics, the driving force behind social media, have shown a strong interest in the growing trend of short video platforms, particularly TikTok. Public reports [11] indicate that younger TikTok users demonstrate a stronger preference for travel compared to older age groups. Despite this, there is limited research systematically exploring the connection between short video platforms and travel preferences, especially among younger users. Generation Z (born 1995–2009) and Generation Y (Millennials, born 1981–1994) are two prominent age groups that dominate social media usage in China, making them the primary TikTok users. Moreover, these two generations also represent a significant portion of the tourism consumer market, using social media to search for travel information and make plans [5]. While public data suggests a higher penetration rate of short videos among these younger groups compared to older generations, empirical studies investigating their behavioral intentions related to travel in the context of short video platforms are still scarce.

Understanding how to tailor video content to potential tourists and identifying the factors that make the video effective, are crucial for both tourist destinations and creators [12]. While recently research has explored various aspects of short-form video as a new entrant in social media marketing communication technology, such as the impact of narrative short videos on customer attitudes toward destination brands [13], the effect of short video advertisements on consumer engagement behavior (Xiao et al., 2023), and the role of short-form videos in addictive behaviors [14]. Most studies have concentrated on user behavior and attitudes towards destinations. Few have specifically examined the impact of short videos on behavioral intentions within the context of tourism [5, 15]. Additionally, studies that investigate TikTok’s influence tend to focus on other sectors, such as education [16] and the cosmetics industry [17]. Although few existing studies focusing on short video on TikTok, such as users’ purchase intentions [18, 19], usage behavior [20], and continuance intention [21]. Therefore, there is a notable gap in the literature regarding the impact of short tourism video platforms’ factors on younger ages’ behavioral intention.

Furthermore, one study applied the UTAUT2 (Unified Theory of Acceptance and Use of Technology 2) model to explore the impact of various motivational factors impact TikTok user’ behavioral intention [5]. Another study focued on the effects of perceived personalization of TikTok content on percived creativity, authenticity, and behavioral intentions, drawing on the Elaboration Likelihood Model [22]. Despite these contributions, the variables considered in prior studies remain limited. To address this gap, this study adopts the S-O-R (Stimulus-Organism-Response) framework, focusing on the influence of the TAM (Technology Acceptance Model) and key external factors on tourists’ flow experience and behavioral intention. In the S-O-R model, the ‘Stimulus’ comprises five external factors that shape tourists’ intentions; the ‘Organism’ refers to internal processes such as telepresence, focused attention, and time distortion; and the ‘Response’ reflects the subsequent behavioral intention. This research thus poses the research objectives (1) to examine the key factors influencing the behavioral intention of young Chinese users when browsing short tourism videos on TikTok; (2) to examine the key factors impacting the flow experience of young Chinese users on TikTok; and (3) to examine the relationship between the flow experience of young Chinese users and their behavioral intention.

The findings of the current study can enhance theoretical and practical developments of knowledge in different ways. On the one hand, it can enrich the tourism literature by exploring the popular topic of short videos. In contrast to previous studies that have primarily analysed short video content, this study considers both technology and content attributes in terms of the emerging TikTok platform. The insights gained from this study will be beneficial to tourist destination managers and social digital marketers. Consequently, the findings can help in adding to the knowledge on the effects of TikTok tourism videos and the impact that they have on the users’ emotions and their behavioral intention

## Literature review

### Stimulus-Organism-Response (S-O-R) model

The S-O-R model was initially proposed by Mehrabian and Russell [23]; it infers that environmental stimulus (S) results in an individual’s emotional response (O), thereby fostering a behavioral response (R). In a short-form video on the TikTok platform, a short tourism video content could be created with different attributes and the unique type of technical in TikTok that act as stimuli to induce the flow experience. In the past, numerous researchers have used flow experience as an organism (O) in different fields [24–26]. The flow experience normally depicts an individual´s psychological or cognitive reaction towards stimuli, but it is the flow experience’s outcome of the reaction stage, that is manifested in the form of the behavioral intention.

The S-O-R (Stimulus-Organism-Response) model has been widely applied in consumer behavior research, particularly to examine offline behavior and online settings [26–28]. While many studies have applied this framework in the tourism context, it has been particularly explored in areas such as smart tourism applications [29, 30], travel booking applications [31, 32], and mobile social tourism platforms for shopping [33].

Social media platforms are widely regarded as useful tools for conveying information to potential tourists and enhancing destination image and behavioral intentions related to travel and recommendations [34]. However, despite this broad understanding, previous studies have paid less attention to clearly identifying the specific attributes and technological factors that drive user behavioral intentions, particularly on short video platforms. TikTok, as a popular new communication platform, has not been comprehensively studied within the tourism sector, particularly in terms of how AI-driven personalized recommendations, automated content generation technology can influence user behavioral intention and tourism decision-marking. Moreover, the research has been limited in articulating how flow experiences as multiple on short video platforms.The current study makes a significant contribution by expanding the application of the S-O-R model. Unlike many studies that rely on the model in a general sense, this research investigates how these specific technological elements evoke flow experiences. These flow experiences, characterized by deep engagement and emotional absorption, are pivotal in shaping users’ behavioral intention to visit a destination or recommend it to others.

To sum up, the current study builds on the S-O-R model to investigate the psychological process by which TikTok’s technology and specific attributes evoke users’ flow experiences, ultimately shaping their behavioral intentions. In this framework, three core aspects of TikTok and two technological factors serve as the Stimuli. The flow experience generated while watching tourism-related videos on TikTok represents the Organism, which, in turn, determines tourists’ behavioral intention—the Response in the model.

### Behavioral intention

Behavioral intention has always been one of the most prominent topics in the tourism industry, acting as a direct determinant of actual behavior [35]. Behavioral intention, as a prediction of individuals’ future behavior, reflects an individual’s expectations and inclinations towards specific behaviors in particular circumstances [35].

Zeithaml et al. [36] is widely recognized as a foundation work in the interpretation of behavioral intention, providing the framework for understanding how intentions influence behavior. More recent studies have emphasized the increasing influence of short videos as new trend application in shaping user behavior intention [5, 37, 38]. For instance, Wang et al. [37] demonstrated that factors and electronic word of mouth associated with short video applications strongly influenced by users’ travel behavior intention. Additionally, other studies have shown that the quality of short video platforms and the influence of social media influencers directly impact their visit intention toward specific destinations [38]. Therefore, short videos have become a crucial factor in shaping tourists’ behavioral intentions. This study reveals that behavioral intention comprises five different dimensions: (1) say positive things about the product or service; (2) recommend the service or product to other customers; (3) remain loyal to the service or product; (4) spend more with service or product; and (5) pay price premiums. Based on this study, numerous subsequent studies by different scholars have investigated the effects of various industries using one or more of the five proposed constructs

### Flow experience

Csikszentmihalyi [39] defined flow as “the holistic sensations that people feel when they act with total involvement”. When someone is in a state of flow, “they become absorbed in their activity” [39]. Flow experience is among the significant theories that has been applied in various fields, such as augmented reality (AR) [40, 41]; online shopping [42]; and education [43]. However, there is less studies related TikTok factors (TAM, entertainment, informativeness, and interactivity) investigate the how short video influence user flow experience.

Although some previous studies have regarded flow as a unidimensional concept [27, 44], many other studies have discussed flow experience as comprising different dimensions in various industries. More detailed information on the flow experience dimensions is provided in Table 1.

**Table 1:** Summary on flow dimensions.

Author and Year	Applications	Construct	Flow Dimensions
Trevino & Webster, 1992 [45]	Human-computer	Flow	Control, attention focus, curiosity, intrinsic interest
Lee & Chen, 2010 [46]	Website	Flow	Concertation, enjoyment, telepresence, time distortion
Zhou et al., 2010 [47]	Mobile social networking	Flow	Perceived enjoyment, perceived control, attention focus
Zhou, 2013 [44]	Mobile communication technology (mobile TV)	Flow	Perceived enjoyment, perceived control, attention focus
Ettis, 2017 [48]	Online store	Flow	Enjoyment, concentration
Jeon et al., 2018 [49]	Website	Flow	Enjoyment, concentration, time distortion
Wu et al., 2020 [50]	Online shopping	Flow	Enjoyment, control, concentration
Kazancoglu & Demir, 2021 [51]	E-retailing	Flow	Enjoyment, goal clarity, curiosity, time distortion, concentration, telepresence, control
An et al., 2021 [24]	Virtual reality (VR)	Flow	Telepresence, focused attention, temporal distortion
Qin et al., 2022 [52]	Social media platform	Flow	Enjoyment, concentration, time distortion

In this current study, three different dimensions of the flow experience are telepresence, focused attention, and time distortion. Telepresence refers to "the degree to which one feels present in a ’virtual space’ rather than in one’s actual physical surroundings" [53]. In other words, the sense of “being there” in an environment mediated by a communication medium is known as telepresence [53, 54]. To varied degrees, all forms of media contribute to the perceptual illusion of being [55]. For instance, users browsing a tourism video may experience a sense of being inside the scene shown in the video. Users’ illusion or feeling is closely related to telepresence [56]. Recent studies have also proven that telepresence is a key component of digital destination marketing [24, 54, 57].

Focused attention is another essential component of flow experience, and it refers to the individual’s focused attention on a particular activity and the degree to which he or she is captivated or mesmerized [58]. As a result of tourists’ flow experience during the viewing of a tourism video via TikTok, their attention becomes highly focused on the virtual environment, thus forcing them to disregard everything that does not fit in the flow experience. Time distortion refers to the degree of users’ feeling “time flying” when they are browsing a tourism video via TikTok. During the flow experience, individuals have a sense of time and perceive it to pass more swiftly than usual [59].

### Technology Acceptance Model (TAM)

The Technology Acceptance Model (TAM) was first developed by Davis in 1989 [60]. In previous studies, the TAM has garnered significant attention, and this theoretical framework has been commonly used to analyze an individual’s acceptance of technologies [61, 62]. According to the TAM, an individual’s behavioral intention to adopt a technology is influenced by two primary beliefs. In support of this assertion, the questionnaire of this study included two factors suggested by Davis [59] perceived usefulness (PU) and perceived ease of use (PEOU). PU is defined as “the degree to which a person believes that using a particular system would enhance his or her job performance”; while PEOU is defined as “the degree to which a person believes that using a particular system would be free of effort” [60].

Numerous scholars have validated the Technology Acceptance Model (TAM) across various sectors, including sports [63, 64], healthcare [65], banking [66], and social media platforms such as YouTube [67]. Building on this foundation, the current study integrates TAM with the Stimulus-Organism-Response (S-O-R) model to offer a more nuanced understanding of how TikTok’s short video platform, augmented by AI-driven technologies, influences tourism-related behavioral intentions. Specifically, AI technologies, such as personalized recommendation and content optimization, play a pivotal role in shaping PU and PEOU by providing more tailored and user-friendly experiences [68]. These AI-enhanced features are integrated into the Stimuli component of the S-O-R model, influencing changes in users’ psychological states (Organism), such as heightened engagement, which subsequently affect their behavioral intentions (Response).

### Relationship between TikTok attributes, TAM, flow and behavioral intention

This study focuses on TikTok technology and attributes, which are PU, PEOU, interactivity, entertainment, and informativeness.

### Technology Acceptance Model (TAM)

As identified in previous literature reviews, many scholars and researchers have advocated the importance of the TAM to study the usage of social media platforms. Indeed, several past studies have established the mediating role of PU, PEOU, and flow experience in these contexts [41, 69]. Furthermore, many researchers have also demonstrated a positive relationship between TAM (PU and PEOU) and telepresence, a key component of flow experience [26, 70]. Zhou [44] established that PEOU is impacted by multiple aspects of flow experience, including attention focus, perceived control, and perceived enjoyment. Hausman and Siekpe [71] found that usefulness influences various factors of flow, such as challenge, concentration, control, and enjoyment. Taken together, PU and PEOU are two crucial factors in TikTok that may impact users’ flow experience. Based on the above discussion, this research proposes the following six hypotheses:

H1a. PU has positive impact on users’ telepresence.
H1b. PU has positive impact on users’ focused attention.
H1c. PU has positive impact on users’ time distortion.
H2a. PEOU has positive impact on users’ telepresence.
H2b. PEOU has positive impact on users’ focused attention.
H2c. PEOU has positive impact on users’ time distortion.

### TikTok attributes

When people are externally stimulated, their feeling of attention is likely to be heightened. Hence, the TikTok platform attributes may have an impact on the subdimensions of flow experience. In the context of social media, attributes such as interactivity, entertainment, and informativeness have been explored separately or partially by various scholars. Wu et al. [50] found that entertainment and informativeness are two key characteristics in online stores, while Sreejesh et al. [72] highlighted interactivity as a crucial factor in social media advertising. However, this study focuses on examining all three elements together within the context of short video platforms.

Previous studies have indicated that social media platform attributes, such as interactivity, entertainment, and informativeness, are important for people’s flow experience [26, 28, 49]. In the context of a two-way communication, the term, ‘interactivity’, can be described as the opportunity of consumers to both receive and actively share some information with its source. When browsing through tourism short videos on TikTok, the audience can click the “like” button, express their opinions to creators, and share the video content with other people. Liu et al. [26] highlighted the importance of interactivity in the social media platform. An investigation into the relationship between the interactivity of the destination marketing organization (DMO) website and flow experience was conducted by Jeon et al. [49]; they proved that interactivity positively impacts on individuals’ flow experience. Furthermore, Arghashi and Yuksel [41] found a positive link between flow experience and interactivity. Therefore, we posit the following hypotheses:

H3a. Interactivity has positive impact on users’ telepresence.
H3b. Interactivity has positive impact on users’ focused attention.
H3c. Interactivity has positive impact on users’ time distortion.

In the realm of social media, entertainment is a pivotal activity that engages users, ensuring they maintain concentration and interest. To a large extent, its purpose is to bring pleasure to or ignite interest of the audience, or to alleviate stress by offering a temporary escape from reality [73]. The contents of short videos are usually entertaining, fun, and enjoyable. Numerous prior studies have found that entertainment has a significantly positive impact on flow experience [28, 74, 75]. For instance, Fahlevi [76] confirmed that entertainment on online travel websites significantly affects flow experience. Hence, this study’s hypotheses is as follows:

H4a. Entertainment has positive impact on users’ telepresence.
H4b. Entertainment has positive impact on users’ focused attention.
H4c. Entertainment has positive impact on users’ time distortion.

Additionally, informativeness is another critically important factor for users’ flow experience on the TikTok platform. Informativeness refers to a website’s ability to give information to its users [77]. Informativeness is typically evaluated in tandem with confirmation that the received information is relevant, up-to-date, timely, and sourced reliably [73]. Many previous researchers and scholars have demonstrated the significant relationship between informativeness and flow experience in different fields [40, 49, 78]. For instance, Yuan et al. [40] demonstrated a positive relationship between the informativeness of AR context and flow experience. Accordingly, the formulated hypotheses for this study are:

H5a. Informativeness has positive impact on users’ telepresence.
H5b. Informativeness has positive impact on users’ focused attention.
H5c. Informativeness has positive impact on users’ time distortion.

### Effects of flow experience

Researchers and scholars have revealed that the state of flow results in an enjoyable experience, and the enjoyment derived from this flow experience encourages users to engage in exploratory activities [79]. Kim (2022) confirmed that users’ behavioral intention on the social media platform is significantly impacted by flow experience. An et al. [25] verified that telepresence in virtual travel experience impacts users’ behavioral intention. Ongsakul et al. [80] found a significantly positive relationship between telepresence in hotel websites and behavioral intention. Chen et al. [81] argued that people who experience flow tend to have a positive relationship with behavioral intention to recommend the destination. Kim & Kim [82] showed that there is a positive correlation between flow experience of the fans and their behavioral intention while watching e-sports live streaming.

**Fig 2:**
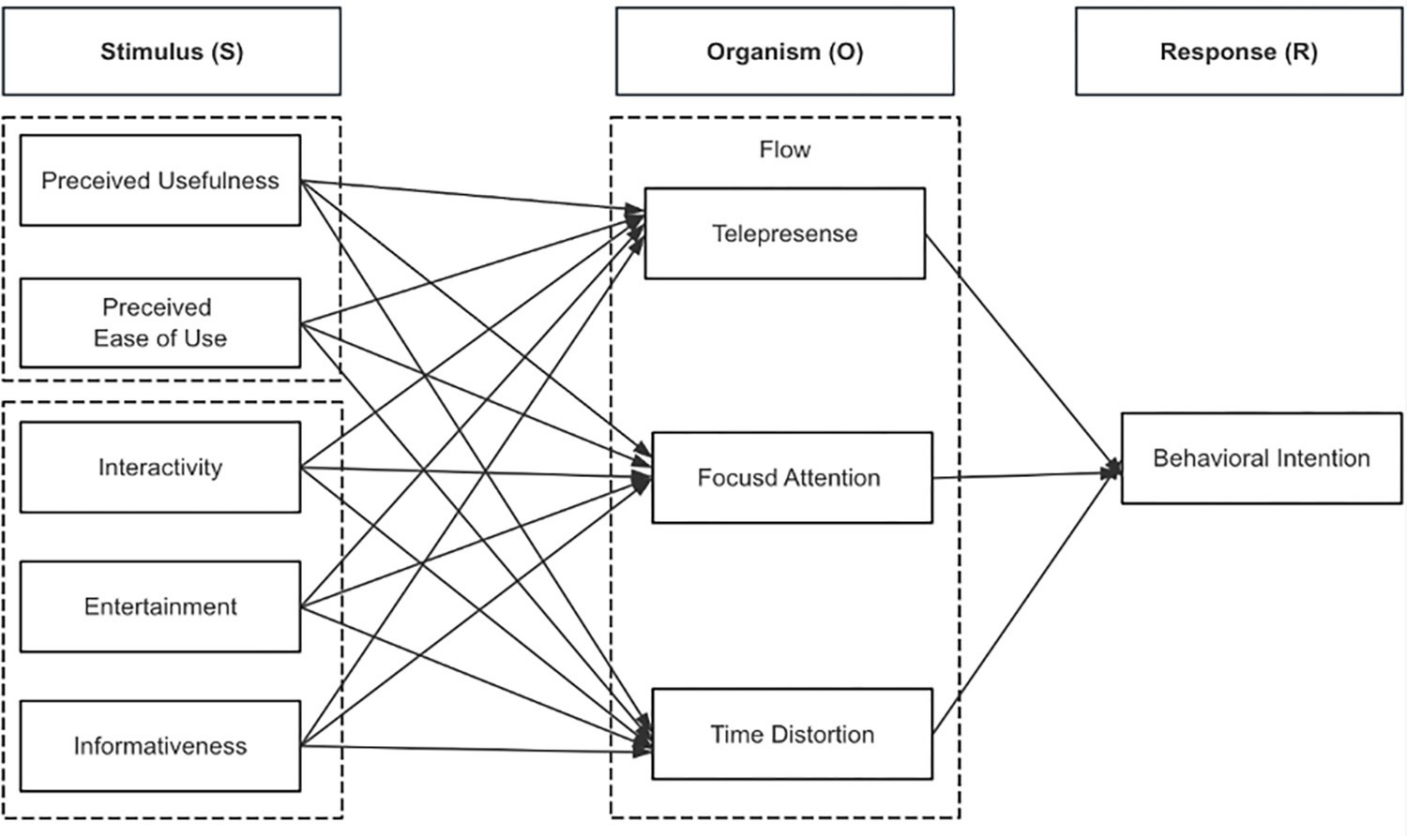
Proposed research model developed by authors. (Source: Own elaboration).

If the platform provides valuable tourism content for users, then flow experience could enhance their behavioral intention on the TikTok platform. Hence, this study assumes that the dimensions of flow experience may play a vital role in shaping users’ behavioral intention. Based on the discussion above, the following research hypotheses are presented below. Fig 2 illustrates the proposed research model of this research.

H6. Telepresence has positive impact on users’ behavioral intention.
H7. Focused attention has positive impact on users’ behavioral intention.
H8. Time distortion has positive impact on users’ behavioral intention.

## Methodology

### Measurement

The current study distributed a questionnaire to individuals who have the habit of browsing short tourism videos on TikTok in China. The technology and attributes of the TikTok platform was conceptualized as PU and PEOU of the TAM, interactivity, entertainment, and informativeness. The TAM was adapted from Chen and Tsai [83], wherein PEOU comprises five items and PU comprises four items. Interactivity was measured by four items adapted from Wang et al. [84]. Both entertainment and informativeness measures included four items each, adapted from Rodgers et al. [85] (2005). For telepresence, five items were utilized from Lee and Chen [46] (2010). Focused attention had four items from previous investigations [44, 86]. Time distortion was measured using three items adapted from Guo and Poole [87]. Lastly, four items of behavioral intention were taken from Choi et al. [88].

Additionally, due to the strong interest exhibited by younger demographics in China toward TikTok, this study specifically focuses on Generation Z and Generation Y as the target groups for analysis. These two age cohorts were selected because of their high levels of engagement with digital and social media platforms, particularly TikTok, where they constitute a significant portion of the user base [89]. The questionnaire for this study was divided into two different sections: (i) the first part gathered information about the respondents using the TikTok platform, such as age and education; and (ii) the second part measured the nine variables in this study. All items on the questionnaire were rated on a five-point Likert scale, with response scores ranging from 1 (strongly disagree) to 5 (strongly agree). The initial questionnaire was developed following modifications based on suggestions and feedback from experts. According to Cooper and Schindler’s [90] study, a pilot test group typically ranges from 25–100 participants. Therefore, this study involved 50 Chinese TikTok users as respondents in the pilot test, and the final questionnaire was refined based on the outcomes and feedback from the pilot test, specifically to validate the measurement scale’s effectiveness and reliability.

### Ethics statement

This study received ethical approval from Universiti Utara Malaysia, ensuring adherence to ethical guidelines for research involving human participants. All participants provided written informed consent prior to their inclusion in the study, after receiving a detailed explanation of the study’s objectives, procedures, potential risks, and their rights as participants. No minors were included in the study, thereby eliminating the need for parental or guardian consent. The informed consent process emphasized participant autonomy and confidentiality, aligning with the ethical standards set forth by the ethics committee.

### Data collection

The data for this questionnaire was collected through a third-party Chinese online survey platform called ’Questionnaire Star’ (Wenjuanxing in Chinese). Wenjuanxing is a tool for designing and distributing questionnaires, similar to platforms like Amazon Mechanical Turk, Survey Monkey, and Survey Gizmo. This survey utilized a cross-sectional design, involving one-time data collection. The questionnaire was administered anonymously to safeguard the confidentiality of respondents’ information. Data was collected from March 11 to March 30, 2024. All 412 respondents are Chinese users of the TikTok platform born between 1981 to 2009. The sample size was determined using G*Power software, which is commonly employed in research to estimate sample size, maximizing statistical power and minimizing the risk of Type II errors. GPower calculates the required sample size based on input parameters such as effect size, alpha level, and power (typically set at 0.80), providing a precise estimation for study design and the tests to be conducted [91]. Further research supports the use of GPower in sample size calculation as it enhances the validity and reliability of outcomes by reducing the likelihood of underpowered or overpowered studies [92, 93].

According to the current model specifications, G*Power calculated a minimum sample size of 138. To ensure a better response rate and robust data, the sample size was increased fourfold, resulting in 552 respondents. Initially, a total of 552 responses were collected. After excluding invalid responses, such as those with unusually fast completion times or pattern-based answers, 412 valid responses were retained for further analysis. Of these respondents, 224 (54.4%) were male and 188 (45.6%) were female; 229 (55.6%) belonged to Generation Z and 183 (44.4%) to Generation Y. The majority of respondents (47.8%) reported using TikTok for one to three years. Table 2 presents the detailed profile of the study’s respondents.

**Table 2:** Profile of this study’s respondents.

Variable		N	%
Gender	Male	224	54.4
	Female	188	45.6
Age	Gen Z (1995–2009)	229	55.6
	Gen Y (1981–1994)	183	44.4
Education level	High school or below	39	9.5
	Vocational college	52	12.6
	Bachelor’s degree	166	40.3
	Master’s degree	120	29.1
	PhD degree	35	8.5
Occupation	Full-time student	49	11.9
	Government agencies/ institutions	68	16.5
	Enterprise staff	115	27.9
	Freelancer	29	7.0
	Self-employment	125	30.3
	Other employees	26	6.3
Monthly income	Below 3000	26	6.3
	3000–5000	51	12.4
	5001–7000	132	32
	7001–9000	128	31.1
	Above 9000	75	18.2
Experience with TikTok	Less than 1 year	114	27.7
	1-3years	197	47.8
	4 years and above	101	24.5

Note: N = 412

### Data analysis

Data were analyzed using SPSS and PLS-SEM 4 (partial least squares structural equation modeling). Descriptive statistics, such as gender and age, were used to profile the respondents. PLS-SEM is particularly well-suited for complex models, does not require the assumption of normal data distribution, and can be effectively applied with smaller sample sizes [94]. The PLS-SEM method is gradually being used in research, especially in marketing and social sciences, due to its capability of analyzing the complex models when the sample size is small, the data is non-normal and when it has the formative constructs. Sarstedt et al. [95] highlighted that PLS-SEM is an ideal method when conducting research to investigate the relationship between the latent variables while achieving reliable and accurate prediction models. PLS-SEM is most applicable in exploratory research or a study whose main objective is theory creation and testing because it can quickly estimate the relationship between several endogenous and exogenous constructs. The analysis followed a two-step approach: first, the measurement model’s reliability and validity were assessed, and then path analysis was conducted to test the hypotheses [96].

### Common method bias

To ensure data accuracy and minimize common method bias, both theoretical and empirical strategies were utilized. Anonymity was preserved, survey items were neutrally phrased, and the question order was randomized to mitigate social desirability and method biases. There are diverse types of variances- CMB is a type of variance that can be mainly attributed to the measurement method employed in the collection of data rather than the actual construct one is measuring [97]. Such kind of methodological biases are especially potential in self-reported surveys, and they raise considerable concerns [97]. One of the concerns when using CMB is that it may introduce systematic measurement biases that may increase or decrease the strength of observed relationships between variables. In order to overcome this, single-factor test by Harman was conducted utilizing the SPSS with unrotated exploratory factor analysis. The analyses revealed that no variable could contribute more than 50% of the total variance; more particularly, one variable could only have 23.41% total variance. This means that common method variance was not a problem in the present research [97].

### Measurement model

The authors first assessed the reliability of each construct by employing both Cronbach’s Alpha (CA) and Composite Reliability (CR), to obtain a thorough evaluation of the consistency of the data. As recommended by previous studies, the value of CA in the social science field should exceed the minimum threshold of 0.7 [98], while if a CR value falls below 0.6, it indicates that the reliability of a construct is relatively weaker and inconsistent [99]. As illustrated in Table 3 provided below, both CA and CR values exceed 0.7, with CA ranging from 0.739 to 0.887, and CR ranging from 0.827 to 0.922.

**Table 3:** Measurement model evaluation.

Constructs	Items	Loadings	CA	CR	AVE	VIF
ENT	ENT1	0.652	0.804	0.872	0.632	2.326
	ENT2	0.825				
	ENT3	0.853				
	ENT4	0.835				
FA	FCA1	0.887	0.881	0.918	0.738	2.004
	FCA2	0.843				
	FCA3	0.840				
	FCA4	0.865				
INF	INF1	0.805	0.805	0.872	0.630	1.989
	INF2	0.783				
	INF3	0.808				
	INF4	0.779				
INT	ITA1	0.865	0.887	0.922	0.747	2.269
	ITA2	0.850				
	ITA3	0.868				
	ITA4	0.875				
PU	PEU1	0.863	0.748	0.827	0.500	1.390
	PEU2	0.798				
	PEU3	0.813				
	PEU4	0.864				
	PEU5	0.756				
PEOU	PRU1	0.720	0.877	0.911	0.672	3.013
	PRU2	0.817				
	PRU3	0.857				
	PRU4	0.551				
	PRU5	0.520				
TEL	TLP1	0.776	0.833	0.882	0.702	2.891
	TLP2	0.654				
	TLP3	0.799				
	TLP4	0.849				
	TLP5	0.787				
TD	TID1	0.803	0.739	0.850	0.655	2.993
	TID2	0.765				
	TID3	0.857				
TBI	TBI1	0.867	0.886	0.921	0.747	3.012
	TBI2	0.879				
	TBI3	0.887				
	TBI4	0.820				

Note: ENT = entertainment, FA = focus attention, INF = informativeness, INT = interactivity, PU = perceived usefulness, PEOU = perceived ease of use, TEL = telepresence, TD = time distortion, TBI = tourists’ behavioral intention.

Convergent validity refers to how various measurements of the same construct demonstrate positive correlations with one another. Convergent validity is typically evaluated using the average variance extracted (AVE) values. The results of this study demonstrate that the values of AVE for all constructs are above 0.5, which are aligned with the recommendation by Fornell and Larcker (1981) for achieving satisfactory convergent validity. As highlighted in Table 3, the AVE values vary from 0.500 to 0.747.

**Fig 3:**
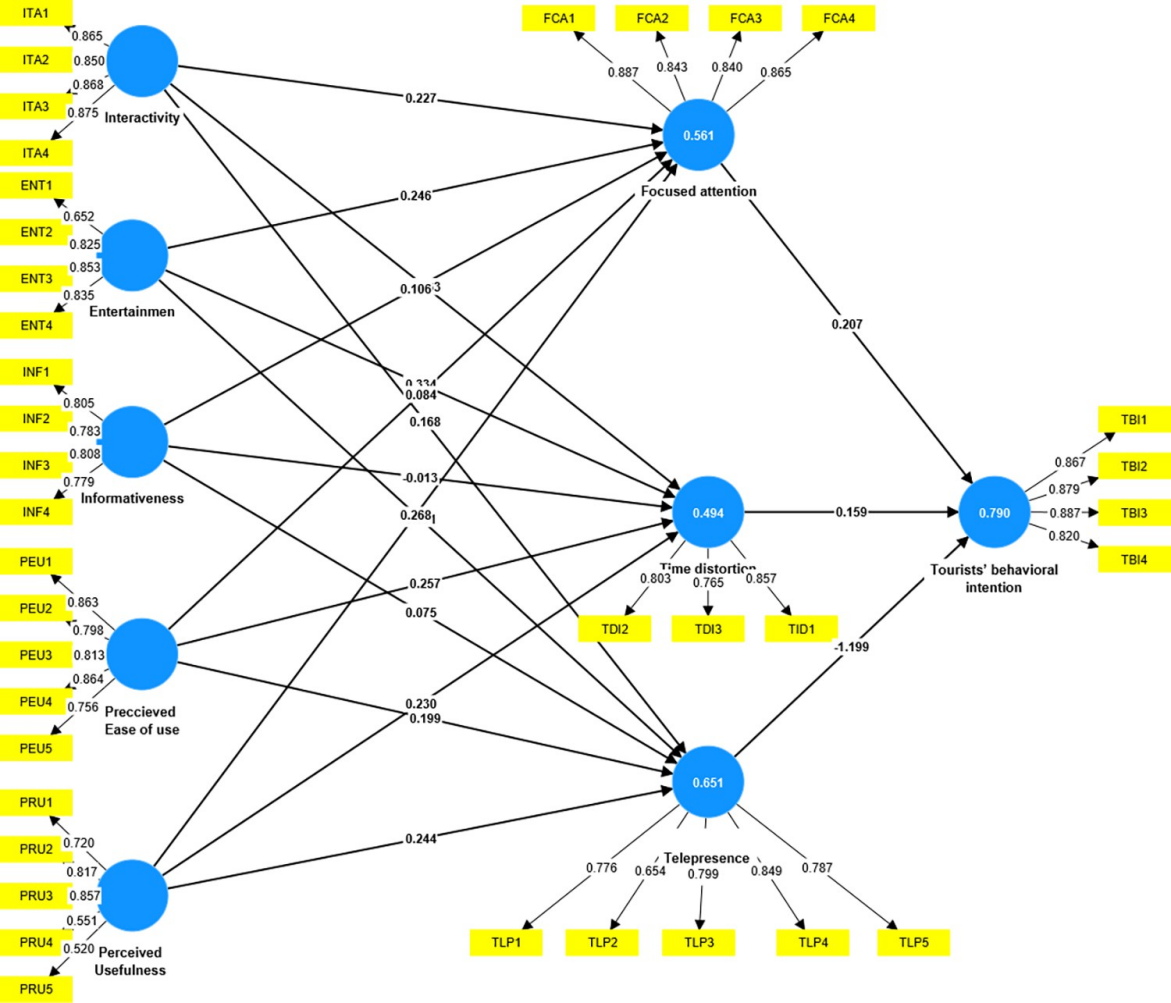
Measurement model (Source: Own elaboration based on smart PLS4).

Hair et al. [99] defined discriminant validity as the “the extent to which a construct is truly distinct from other constructs by empirical standards”. The Fornell-Larker criteria and Hetrotrait-Monotrait (HTMT) correlation ratio are both methods used to assess discriminant validity, ensuring that each construct in the measurement model is distinct from others. As presented in Tables 4 and 5 all of the obtained values are below 0.9, indicating that each of the constructs is different from the other. The study results demonstrate that discriminant validity is fully achieved. Fig 3 represents the measurement model of this study.

**Table 4:** Fornell-Larcker criterion.

	1	2	3	4	5	6	7	8	9
ENT	0.795								
FA	0.625	0.859							
INF	0.688	0.543	0.794						
INT	0.531	0.613	0.431	0.865					
PU	0.550	0.650	0.516	0.679	0.706				
PEOU	0.464	0.439	0.388	0.336	0.464	0.820			
TEL	0.699	0.770	0.578	0.608	0.671	0.551	0.876		
TD	0.604	0.618	0.462	0.477	0.569	0.535	0.850	0.889	
TBI	-0.721	-0.738	-0.579	-0.608	-0.649	-0.604	-0.784	-0.733	0.863

**Table 5:** HTMT criterion.

	1	2	3	4	5	6	7	8	9
ENT									
FA	0.736								
INF	0.870	0.643							
INT	0.630	0.692	0.507						
PU	0.692	0.758	0.675	0.776					
PEOU	0.554	0.499	0.460	0.382	0.621				
TEL	0.840	0.719	0.703	0.705	0.809	0.641			
TD	0.756	0.743	0.586	0.570	0.714	0.654	0.778		
TBI	0.847	0.835	0.685	0.688	0.762	0.683	0.601	0.881	

### Hypotheses testing

First, the authors analyzed the effects of the external factors of focused attention, telepresence, time distortion, and tourists’ behavioral intention. A bootstrapping with a sample size of 5000 and a significance level of 5% was applied to assess the path’s significance. Further, the authors investigated the structural model’s parameters. The coefficient of determination (R^2^) quantifies the variation in the endogenous variable that is explained by the independent variable constructs [100]. Chin [101] demonstrated that R^2^ values of approximately 0.19, 0.33, and 0.67, are generally considered as indicating weak, moderate, and substantial relationships, respectively. In the current study, the adjusted R^2^ values for telepresence, focused attention, time distortion, and tourists’ behavioral intention are 0.555, 0.646, 0.488, and 0.789, respectively, indicating that the structural model has a strong prediction ability for these constructs.

Table 6 presents the results of the current study’s proposed model, including standard deviations and t-values. The findings indicate that PU has a significantly positive impact on telepresence (t = 6.030, p<0.000), focused attention (t = 6.398, p<0.000), and time distortion (t = 4.014, p<0.000). Hence, H1a, H1b, and H1c are supported. Moreover, PEOU positively influences telepresence (t = 5.805, p<0.000), focused attention (t = 2.371, p<0.000), and time distortion (t = 5.805, p<0.000), supporting H2a, H2b, and H2c. Interactivity positively influences telepresence (t = 4.086, p<0.000) and focused attention (t = 4.793, p<0.000), but the impact of time distortion is insignificant. Hence, H3a and H3b are supported but H3c is not supported. In addition, entertainment positively impacts telepresence (t = 6.134, p<0.000), focused attention (t = 4.063, p<0.000), and time distortion (t = 5.626, p<0.000). Therefore, H4a, H4b, and H4c are all supported. Informativeness positively impacts focused attention (t = 1.727, p<0.000), but is insignificant in telepresence and time distortion. Therefore, H5b is supported, but H5a and H5c are not supported. Focused attention (t = 3.331, p<0.000) and time distortion (t = 3.115, p<0.001) positively impact tourists’ behavioral intention, but telepresence shows an insignificant result for tourists’ behavioral intention. Therefore, H7 and H8 are supported but H6 is not supported.

**Table 6:** Hypothesis testing.

Path	SD	T-value	P-value	results
PU -> TEL	0.040	6.030	0.000	Significant
PU ->FA	0.042	6.398	0.000	Significant
PU -> TD	0.057	4.014	0.000	Significant
PEOU -> TEL	0.034	5.805	0.000	Significant
PEOU-> FA	0.035	2.371	0.009	Significant
PEOU -> TD	0.044	5.805	0.000	Significant
INT -> TEL	0.041	4.086	0.000	Significant
INT -> FA	0.047	4.793	0.000	Significant
INT -> TD	0.048	1.303	0.097	insignificant
ENT ->TEL	0.054	6.134	0.000	Significant
ENT -> FA	0.061	4.063	0.000	Significant
ENT -> TD	0.059	5.626	0.000	Significant
INF -> TEL	0.055	1.372	0.085	insignificant
INF -> FA	0.061	1.727	0.042	Significant
INF-> TD	0.060	0.211	0.417	insignificant
TEL->TBI	0.083	14.433	0.000	insignificant
FA->TBI	0.062	3.331	0.000	Significant
TD->TBI	0.051	3.115	0.001	Significant

### PLS-Predict

The degree of fit of a structural model for prediction is conventionally evaluated based on Stone-Geisser’s Q^2^, which is calculated using the blindfolding technique [96]. This technique excludes every dth data point to forecast the excluded sections and make sure that d is chosen in such a way that the ratio of the valid observation to d is not integral. The authors concluded that the cross-validated redundancy measure is preferable for PLS-SEM since it takes into account both, the structural and measurement models [94]. The Q^2^ value above zero suggests a measure of predictive relevance for the model. In the current study all the Q2 values of endogenous constructs are greater than zero.

## Discussion

In the era of digital innovation, short-form videos are a key method for building a bridge of communication between destinations and consumers [5]. This study, based on the S-O-R model, investigated how flow experience impacts TikTok attributes and technology factors on tourists’ behavioral intention within the context of China’s tourism through the TikTok platform. Although many prior tourism studies have focused on flow experience as a unidimensional construct [26, 81], this study takes a novel approach by examining flow experience as a multi-dimensional construct, primarily focusing on three key dimensions: telepresence, focused attention, and time distortion.

Of the 18 hypotheses, this study finds support for 14. Firstly, highlighting several significant relationships. Firstly, the findings indicate that PU and PEOU positively affect an individual’s flow experience. This suggests that the TAM framework within the platform can directly enhance the young users’ flow experience (telepresence, focused attention, and time distortion) while browsing short tourism videos on TikTok. These findings align with recent research, such as Chen et al. [42] and Hyun et al. [69]. While these relationships are not entirely new, their influence within the tourism sector on TikTok remains underexplored. Additionally, this study empirically confirms the positive impact of entertainment and flow experience, supporting the notion that engaging content on TikTok enhances users’ enjoyment and immersion.

Second, interactivity had significant relationships with focused attention and telepresence. The results are consistent with Liu [102], who also showed a positive link between informativeness and focused attention. Although some studies have shown that informativeness can generate telepresence and time distortion in certain settings [26, 49], this study proves that informativeness is negatively linked to telepresence and time distortion. The results suggest that information overload and diminished entertainment value may be affecting younger users’ experiences. Although accurate, timely, complete, and up-to-date information is valuable, it can detract from younger audiences’ flow experience if an abundance of information overwhelms or diverts their attention from the more engaging and visually appealing elements of video content. For these users, who generally prefer concise and visually stimulating material, an overload of detailed tourism information may reduce their overall enjoyment and sense of immersion.

Additionally, focused attention and time distortion are two key factors influencing tourists’ behavioral intention. However, the results of this study do not demonstrate a relationship between telepresence and tourists’ behavioral intention. While previous scholars have found evidence that telepresence positively impacts behavioral intention, our findings do not support this in the context of short tourism videos on TikTok. A review of prior research suggests that the direction of causality between telepresence and tourists’ behavioral intention remains uncertain. This study identifies a significant and testable phenomenon in which tourists’ flow experience fluctuates during TikTok activity. In conclusion, although telepresence can generate a strong sense of immersion and escapism, this does not necessarily translate into tourists’ behavioral intention, likely due to the temporary, virtual nature of the experience and its limited connection to real-world decision-making factors.

### Theoretical implications

This study provides several theoretical contributions to the academic fields of tourism and short video marketing. First, tourism is really important to national economies as it greatly contributes to economic growth and the creation of new jobs. However, most of the previous research has failed to consider the convergence of tourism with the short video platforms, and specifically exclude the massive and powerful younger generation. To fill this gap, this study provides insights into how short videos, such as those featured in TikTok, impact the tourism choices of the youth.

Secondly, the findings indicate the suitability of the S-O-R model for understanding tourists’ psychological responses to short videos within the TikTok tourism environment. The S-O-R model depicts how stimuli (S) which affect the internal workings of organisms (O), and subsequently, elicit specific responses (R), are used to frame the stimuli in terms of the attributes of TikTok content and the enabling technology. The findings show that elements, such as video quality, content relevance, and technological interactivity, act as organizing stimuli that activate a range of processes within the mind of a tourist. These internal processes enable the psychological state of flow among the tourists, and consequently, affect their behavioral intention, as to how they engage and decide to visit a particular destination portrayed in the videos.

Lastly, this study offers an exploratory investigation into the multifaceted construct of flow experience in tourism and short video applications. Research on flow experience has been done from various viewpoints, such as perceived control, perceived enjoyment, skills, and challenges [44, 52, 103]. This research builds upon these dimensions by determining the roles of time distortion and focused attention in stimuli and behavioral intention. The findings also suggest that these dimensions of flow experience play an important role in the behavioral intention of tourists. In more detail, when consumers feel that time is passing quickly and pay particular attention to TikTok videos, it is possible to develop a clear intention to visit the places depicted in the videos.

### Practical implications

The present research paper offers the following research contributions that require further discussion when it comes to applicable practical implications for practitioners and policymakers within the tourism and digital marketing fields. First, the results point out that destination marketers must understand and address all the challenges of creating short promotional videos that would feature whipped visuals and optimal sound. Such videos should be shot with high videography standards and be able to capture the tourists’ attention from the initial glance. This is important because first impressions of a video that has been produced can be critical determining factors of people’s reactions and activities later on.

From a quantitative standpoint, the research demonstrates that there are significantly moderate and positive relationships between the quality of short promotional videos and flow experience. The findings may be useful to government policymakers. In particular, the study focuses on the impact of short tourism videos that go viral on TikTok for decision-making about travel, offering policymakers a roadmap to using these videos to promote domestic tourism destinations. In other words, through well-organized advertisement campaigns employing short videos that capture local attractions, governments may increase tourist revenues. For example, creating quality short tourism videos may lead to the creation of curiosity among tourists to visit specific local destinations, to discover such areas.

In addition, the study emphasizes that the correlations found require the TikTok developers to increase the level of platform attributes, especially for informativeness and interactivity, to improve the flow experience for the users. From the data presented in this study, it is apparent that while telepresence might help to boost interest, it might hinder tourists’ behavioral intentions if not well addressed. Thus, it will be necessary to work with the TikTok developers to enhance features of the application for better telepresence in short videos. Telepresence can be defined as the further augmentation of the experience of viewing television; the increased engagement of the senses can be accomplished by increasing the quality of the picture and incorporating other features, such as interactivity or the incorporation of information.

In the same way, the findings of the study signify the need to foster innovation in the creation of digital content and innovations in online platforms. The challenge for destination marketers is not only to stay abreast of current trends, but to also determine prospective future evolutions in digital marketing strategies. Thus, marketers can be one step ahead of the competition and develop more enticing and efficient promotional materials for the target group interested in travelling. The findings of this study therefore bring significant implications to policymakers regarding the strategic management of tourism through the effective use of social media to complement traditional marketing tools. Using Instagram, for example, or TikTok that has millions of daily active users, the policymakers can ensure that people are informed of the lesser-known travel destinations. This can help to provide a broader distribution of tourism geographically, which may help to reduce overcrowding in certain regions, while also assisting other regions that have not been as heavily visited to develop their economies through tourism.

### Limitations and future research

The limitations and future directions of the study are presented which can help the readers to focus on further research. Firstly, this research focused only on Generations Z and Y in Mainland China. While this methodology is useful to decode the attitudes and actions exhibited by these groups, it may not present the realities of the other generations, like the Generation X or Baby Boomers or positions of different regions in China. It is suggested that further research should cover people of various ages and from different locations in China to provide a broader perspective and identify nation-wide patterns.

The study only focused on the Chinese version of TikTok or Douyin, which may limit the generalization of the results. Since there is an existence of various other short video platforms in the world today, future researchers should consider other platforms, such as Instagram Reels, YouTube Shorts, and Snapchat, among others. In turn, it would help in the conduct of a comparative analysis after examining different platforms and regions, and hence, deepen the existing understanding of short video marketing trends.

In evaluating the research, the methodological type was quantitative, given that data was collected through a questionnaire survey. Although this method generates descriptive statistics, which are useful to understand participants’ phenomena, and to explore the relationships between them, it does not offer a detailed interpretation of participants’ experiences and motivations. Further studies based on the present research may use other methodological approaches, which can include the use of questionnaires, interviews, focus group discussions, and content analysis. This would also help in extending research on short video marketing in China’s tourism industry where there is adequate evidence of preferences, emotions, and even perceptions.

In addition, this study used a cross-sectional design, whereby data were collected during one point. In time. Under this approach, there is no way of gauging changes and developments based on current trends and existing relationships. Future researchers could use a more complex method, for example, a longitudinal research design and using data collection in multiple time points. This would give more ideas on the different ways that short video marketing strategies can turn out and the relationship of users’ engagement and preferences over time, thus giving more real-time insights.

Finally, it is determined that utilizing short videos as a form of marketing involves some limitations that should be taken into account by those who plan on conducting future research on this topic, particularly the potential role of socio-cultural and economic factors. For example, exploring the relationship between culture and the strategies and content utilized in short videos via these platforms, or exploring the relationship between economic status and device access and the behaviors exhibited on these platforms, would enhance the understanding of the environment in which these platforms exist. In future studies, the present findings can be enriched and extended, which will provide a more detailed conclusion of short video marketing in China’s tourism industry, and even the global tourism industry.

## Supporting information

S1 File Measurement items. (DOCX)

S2 File Data. (XLSX)
