# Supplementary material for: The impact of TikTok short video factors on tourists’ behavioral intention among Generation Z and Millennials: The role of flow experience
Source: PLoS One. 2024 Dec 5;19(12):e0315140. doi: 10.1371/journal.pone.0315140 (PMC11620689; doi:10.1371/journal.pone.0315140)
Supplement: S1 File — (DOCX) [file pone.0315140.s001.docx]

**Supporting Information**

**S1. Measurement items**

| Items |
| --- |
| Perceived Ease of Use (PEOU)  PEOU1. I think I can easily operate this app.  PEOU2. I can very quickly learn how to operate this app.  PEOU3. I can very quickly operate this app to get information I need.  PEOU4. Overall, I think this app is easy to use.  PEOU5. I think the operation interface of this app is easy and straightforward.  Perceived of Usefulness (PU)  PU1. I think using this app can help me obtain tourism information I need.  PU2. I think using this app can increase the efficiency of tourism information collection.  PU3. I think how app incorporated tourism video helps me in tour planning.  PU4. Overall, I think using this app is useful.  Interactivity  INT1. When I am browsing tourism video on TikTok, I can interact and communicate with the publishers on time.  INT2. When I am browsing tourism video on TikTok usually inspires me to share the video link to other people.  INT3. When I am browsing tourism video on TikTok, I would like to express my opinions and comments in the discussion forum.  INT4. When I am browsing tourism video on TikTok, I can get quick feedback from the publisher on my questions.  Informativeness  INF1. TikTok provides me accurate source of tourism information.  INF2. TikTok provides me timely tourism information.  INF3. TikTok provides up-to-date tourism information.  INF4. TikTok supplies complete tourism information.  Entertainment  ENT1. Watching short tourism video on TikTok is entertaining.  ENT2. Watching short tourism video on TikTok is enjoyable.  ENT3. Watching short tourism video on TikTok is pleasing.  ENT4. Short tourism video on TikTok fun to watch.  Telepresence  TEL1. I forget about my immediate surroundings when I browsing tourism video on TikTok platform.  TEL2. Browsing the tourism video on TikTok often made me forget where I was.  TEL3. When I browsing tourism video on TikTok, I felt I was in a world created by the videos I visited.  TEL4. After browsing tourism video on TikTok, I felt like I came back to the “real world” after a journey.  TEL5. When I browsing tourism video on TikTok, the world generated by the website I visited was more real for me than the “real world”.  Focused attention  FA1. When browsing tourism video on TikTok, I am absorbed intensely in the activity.  FA2. When browsing tourism video on TikTok, my attention is focused on the activity.  FA3. When browsing tourism video on TikTok, I concentrate fully on the activity.  FA.4 When browsing tourism video on TikTok, I am deeply engrossed in the activity.  Time distortion  TD1. Time seems to go by very quickly when I browsing tourism video on TikTok.  TD2. When I browsing tourism video on TikTok, I tend to lose track of time.  TD3. When I browsing tourism video on TikTok, I forgot the time flow.  Behavioral intention  BI1. I would recommend the tourist destination I viewed on the TikTok to a friend.  BI2. I would say positive things about the tourist destination  BI3. I would visit the tourist destination I viewed on the TikTok.  BI4. I would seek more information about the tourist destination I viewed on the TikTok. |
